# A novel salt- and organic solvent-tolerant phosphite dehydrogenase from *Cyanothece* sp. ATCC 51142

**DOI:** 10.3389/fbioe.2023.1255582

**Published:** 2023-08-18

**Authors:** Gamal Nasser Abdel-Hady, Takahisa Tajima, Takeshi Ikeda, Takenori Ishida, Hisakage Funabashi, Akio Kuroda, Ryuichi Hirota

**Affiliations:** ^1^ Unit of Biotechnology, Division of Biological and Life Sciences, Graduate School of Integrated Sciences for Life, Hiroshima University, Hiroshima, Japan; ^2^ Department of Genetics, Faculty of Agriculture, Minia University, Minia, Egypt; ^3^ Seto Inland Sea Carbon-neutral Research Center, Hiroshima University, Hiroshima, Japan

**Keywords:** phosphite dehydrogenase, NAD(P)H regeneration, salt resistance, organic solvent tolerance, low water activity

## Abstract

Phosphite dehydrogenase (PtxD) is a promising enzyme for NAD(P)H regeneration. To expand the usability of PtxD, we cloned, expressed, and analyzed PtxD from the marine cyanobacterium *Cyanothece* sp. ATCC 51142 (Ct-PtxD). Ct-PtxD exhibited maximum activity at pH 9.0°C and 50°C and high stability over a wide pH range of 6.0–10.0. Compared to previously reported PtxDs, Ct-PtxD showed increased resistance to salt ions such as Na^+^, K^+^, and NH_4_^+^. It also exhibited high tolerance to organic solvents such as ethanol, dimethylformamide, and methanol when bound to its preferred cofactor, NAD^+^. Remarkably, these organic solvents enhanced the Ct-PtxD activity while inhibiting the PtxD activity of *Ralstonia* sp. 4506 (Rs-PtxD) at concentrations ranging from 10% to 30%. Molecular electrostatic potential analysis showed that the NAD^+^-binding site of Ct-PtxD was rich in positively charged residues, which may attract the negatively charged pyrophosphate group of NAD^+^ under high-salt conditions. Amino acid composition analysis revealed that Ct-PtxD contained fewer hydrophobic amino acids than other PtxD enzymes, which reduced the hydrophobicity and increased the hydration of protein surface under low water activity. We also demonstrated that the NADH regeneration system using Ct-PtxD is useful for the coupled chiral conversion of trimethylpyruvic acid into *L*-tert-leucine using leucine dehydrogenase under high ammonium conditions, which is less supported by the Rs-PtxD enzyme. These results imply that Ct-PtxD might be a potential candidate for NAD(P)H regeneration in industrial applications under the reaction conditions containing salt and organic solvent.

## 1 Introduction

Enzymes can potentially play crucial roles in the development of sustainable bioconversion processes in chemical, biotechnological, agricultural, and pharmaceutical industries (Kumar and Sharma, 2016; Chapman et al., 2018). Over the last few decades, thousands of new enzymes have been discovered and used as industrial catalysts (Deng et al., 2019). Their high catalytic activity, mild reaction conditions, and remarkable substrate specificity make them highly efficient biocatalysts for industrial-scale bioprocessing (Madhavan et al., 2017; Roy and Prasad, 2017). With recent developments in genetic and protein engineering technologies, the number of enzymes has increased, and they are being tailored as specific biocatalysts for bioproduction. However, the overall number of industrial applications of these enzymes remains limited, mostly because of the absence of good operational stability under unusual conditions, such as the presence of high salt and organic solvents and a lack of catalytic activity at high or low temperatures (Elleuche et al., 2014; Reetz, 2016; Chapman et al., 2018).

Salt- and organic solvent-tolerant enzymes are attractive for biotechnological applications because of their ability to open new opportunities for enzyme-based industrial processes. Water activity, which is a measure of the water content surrounding enzyme molecules, significantly influences catalytic activity in the presence of salts or organic solvents (Kikani et al., 2023). High salinity and organic solvents disrupt water structure, thereby limiting free water for enzyme hydration, with deleterious effects on enzyme structure and activity (Persson and Halle, 2008; Karan et al., 2012). However, enzymes that are stable in salt or organic solvents can be activated by maintaining a tight hydration shell under low water activity and expanding their range of application under extreme conditions (Karan et al., 2012). Furthermore, bioconversion in organic solvents offers several distinctive industrial advantages, such as improving the solubility of nonpolar substrates that are relatively insoluble in water (Khmelnitsky et al., 1988). The rate of mass transfer during dissolution is influenced by the solubility of the substrate, meaning increased substrate solubility affords rapid mass transfer and reaction rates for hydrophobic compounds (Doukyua and Ogino, 2010). These organic systems are advantageous for enzymes that synthesize esters and peptides because of the change in the thermodynamic equilibria in favor of synthesis over hydrolysis (Elmore, 2002; Kumar and Bhalla, 2005). Organic solvent systems can empower chemical reactions that are not feasible in aqueous media and reduce the risk of microbial contamination in the reaction solution (Carrea and Riva, 2000; Klibanov, 2001; Schmid et al., 2001). However, enzymatic reactions under low water activity conditions are restricted because of the insufficient activity and stability of most enzymes in the presence of salts or organic solvents (Doukyua and Ogino, 2010). Currently, protein engineering-based methods, such as site-directed mutagenesis, gene shuffling, and directed evolution, have been developed to overcome this limitation and improve the activity and stability of enzymes under high salt or organic solvent conditions (Iyer and Ananthanarayan, 2008; Kaul and Asano, 2011). However, these approaches are often laborious, lengthy, and do not always result in successful outcomes because improved enzyme stability mostly generates from specific mutations, which typically do not follow any evident trends or patterns (Coker, 2016; Jimenez-Rosales and Flores-Merino, 2018). Therefore, it is important to obtain native salt- and organic solvent-active enzymes that can be used without any modification for practical applications that employ high salt or organic solvents as reaction media.

Phosphite dehydrogenase (PtxD), which was initially isolated from *Pseudomonas stutzeri* WM88 (Ps-PtxD), catalyzes the oxidation of phosphite (Pt) into phosphate (Pi) coupling with NAD^+^ reduction into NADH (Costas et al., 2001). Following this report, a variety of bacteria dwelling in soil and aquatic environments have been found to encode PtxD enzymes (White and Metcalf, 2007; Hirota et al., 2012). In addition, owing to the increased availability of genomic information, homologous *ptxD* genes were found in several microbial genomes. As compared to other NAD(P)H regeneration systems such as formate dehydrogenase (FDH) and glucose dehydrogenase (GDH), PtxD has practical value as an NAD(P)H cofactor-regeneration enzyme in biotechnology due to the substantial change in free energy of this reaction (Δ*G°*′ = −63.3 kJ/mol) and a high equilibrium constant (*K*_eq_ = 1 × 10^11^). PtxD utilizes an inexpensive Pt substrate that is produced as a by-product of several industrial processes (Kuroda and Hirota, 2015). Furthermore, the phosphate produced via phosphite oxidation acts as a buffer and can be readily separated by calcium precipitation if required (Vrtis et al., 2002; Relyea and van der Donk, 2005). Therefore, PtxD has received considerable research attention as an alternative system for the regeneration of cofactor NAD(P)H. Previously, we isolated Rs-PtxD from a soil bacterium, *Ralstonia* sp. 4,506, which shows higher catalytic activity (*k*_cat_/*K*_M_^NAD^, 16.6 at 37°C) and an extended half-life at 45°C (80.5 h) than that of Ps-PtxD (Hirota et al., 2012; Abdel-Hady et al., 2021). Furthermore, we recently created Rs-PtxD mutants via site-directed mutagenesis with higher catalytic efficiency toward NADP^+^, which expanded its application in NAD(P)H regeneration in industrial applications (Abdel-Hady et al., 2021). However, PtxDs are susceptible to salt and organic solvents, limiting their practical application (Costas et al., 2001; Hirota et al., 2012).

In the present study, we cloned a PtxD from *Cyanothece* sp. ATCC 51142 (Ct-PtxD) (*Cyanothece* 51142, recently reclassified as *Crocosphaera subtropica*; Mareš et al., 2019), which exhibited higher salt resistance and organic solvent tolerance. Biochemical analysis revealed that Ct-PtxD enzyme exhibited not only high salt resistance and organic solvent tolerance, but also enhanced activity in the presence of organic solvents. The applicability of Ct-PtxD as an NADH regeneration system was demonstrated during the conversion of trimethylpyruvic acid (TMP) into *L*-tert-leucine by leucine dehydrogenase (LeuDH) under high ammonium conditions.

## 2 Material and methods

### 2.1 Screening of *ptxD* genes

The Rs-PtxD was obtained from the soil bacterium *Ralstonia* sp. 4506 (GenBank accession number: BAV60262) (Hirota et al., 2012). The amino acid sequence of Rs-PtxD was used as a query in BLAST to search for homologous phosphite dehydrogenase proteins. Potential genes encoding phosphite dehydrogenase are mainly found in the bacterial genome in unidentified sequences. A phylogenetic tree of the potential phosphite dehydrogenase was constructed using ClustalX2 and MEGA-X (Supplementary Figure S1). According to phylogenetic tree analysis, PtxD derived from *Cyanothece* sp*.* ATCC 51142 (59.8% amino acid identity with Rs-PtxD) was selected. Bacterial strains and genomic DNAs were obtained from domestic and foreign microbial strain-preservation institutions (ATCC, NBRC, and JCM). The accession numbers of the PtxD proteins used in this analysis are shown in Supplementary Figure S1.

### 2.2 Cloning of the *ptxD* gene

The *ptxD* gene was amplified from *Cyanothece* sp. ATCC 51142 strain (Ct-PtxD) using primers 5′-GGC​CGC​ATA​TGA​ATC​AAA​AAC​CTA​AAG​TTG-3′ and 5′-TTA​TTC​TCG​AGT​ACG​ATA​CCA​TTT​ACA​GCA​C-3′, with genomic DNA obtained from the American Type Culture Collection (ATCC) as the template DNA. PCR was performed with primers under the following conditions: 98°C for 10 s, 58°C for 30 s, and 68°C for 30 s for 30 cycles. The purified PCR product was ligated into pET-21b (+) digested with *Nde*I and *Xho*I restriction enzymes using an In-Fusion HD cloning kit (Takara Bio Inc., Shiga, Japan). The DNA fragments cloned into the vector plasmid were verified by sequencing.

### 2.3 Protein expression and purification of PtxD protein

For Ct-PtxD protein expression, *Escherichia coli* Rosetta2 (DE3) pLysS (Novagen) harboring *Ct-ptxD/*pET-21b (+) were grown in the 2×YT liquid medium (Sambrook et al., 1989) at 37°C overnight. Thereafter, 50 mL of fresh medium was inoculated with 1% (v/v) cell from an overnight culture and allowed to grow at 37°C until an OD_600_ of 0.5. The culture was induced by the addition of 0.2 mM isopropyl *β*-D-thiogalactopyranoside (IPTG) at 28°C for another 6 h. The cells were precipitated by centrifugation and resuspended in 5 mL of 20 mM Tris-HCl (pH 7.4), 50 mM NaCl, and 20% glycerol, and disrupted on ice using a probe sonicator (Branson Sonifier, 4 min with a pulse sequence of 1 s on and 2 s off at 20% amplitude level). The crude extracts were clarified by centrifugation at 20,000 *× g* for 30 min at 4°C. The resultant supernatant was filtered through a 0.45-μm filter and loaded onto a 1-mL HisTrap FF column (GE Healthcare United Kingdom Ltd., Little Chalfont, United Kingdom) equilibrated in buffer A (20 mM Tris-HCl [pH 7.4], 50 mM NaCl, and 20% glycerol). After binding of the Histidine-tagged recombinant proteins, the column was washed with 10 mL buffer A and eluted with a linear 10-mL gradient of imidazole (0–0.5 M) in buffer A. The fractions containing Ct-PtxD were combined, and the buffer solution was substituted to 50 mM Tris-HCl (pH 7.4) and 15% glycerol by ultrafiltration using Amicon Ultra centrifugal filtration devices (10 kDa molecular mass cutoff) (Merck Millipore, Darmstadt, Germany). The concentrations of purified protein were determined using the Bradford method (Bradford, 1976) with bovine serum albumin (BSA) as a standard.

### 2.4 PtxD activity assay and kinetics of Ct-PtxD

Phosphite dehydrogenase activity was assayed by using a reaction mixture containing 1 mM Pt, 20 mM morpholinepropanesulfonic acid (MOPS, pH 7.3), 0.5 mM NAD(P)^+^, and 2.0 μg of PtxD in the total reaction volume of one milliliter. Prior to the reaction, the reaction mixture without containing Pt was incubated at 37°C for 2 min. Then, the reaction was started by adding Pt solution and running for 10 min at 37°C. Initial velocities were determined by measuring the increase in absorbance at 340 nm using a Beckman DU-800 spectrophotometer (Beckman Coulter, CA, USA), and a molar extinction coefficient of 6,220 M^-1^ cm^-1^ was used to calculate NAD(P)H production (Bucher et al., 1974). One enzyme unit was defined as the amount of enzyme that catalyzed the formation of 1 µmol of NAD(P)H per minute under the conditions described above.

Assay investigating Ct-PtxD kinetics was conducted in solutions containing Pt, NAD(P)^+^, 5.0 μg/mL Ct-PtxD protein, and 100 mM MOPS (pH 7.3). The kinetic parameters of one substrate were determined by varying its concentration, whereas holding the other substrate at saturating concentration (1.0 mM). The initial rates were determined using a Beckman DU-800 spectrophotometer to monitor the increase in NAD(P)H formation at 340 nm. The kinetic parameters, *K*_M_ and *k*_cat_, for NAD(P)^+^ and Pt were calculated using Lineweaver–Burk plots.

To assess the effect of nicotinamide cofactor (NAD^+^/NADP^+^) binding with PtxD on thermostability and organic solvent stability, a solution containing 0.2 μg/μL PtxD protein in 50 mM MOPS (pH 7.3) was preincubated on ice with or without 0.5 mM NAD(P)^+^ for 5 min and used for measuring the enzymatic activity.

### 2.5 Effects of pH, optimal temperature, and NAD(P)^+^ binding on Ct-PtxD

The optimum pH was estimated by measuring Ct-PtxD activity under varying pH conditions. The MOPS buffer (pH 7.3) in the reaction mixture was substituted with the following buffers: 2-morpholinoethanesulfonic acid (MES) buffer for pH 6.0–7.0, MOPS buffer for pH 7.0–8.0, Tris-HCl for pH 8.0–9.0, and N-cyclohexyl-2-aminoethanesulfonic acid (CHES) for pH 9.0–10.0 at a final concentration of 20 mM. The PtxD activity was determined as described above. The pH stability profile of Ct-PtxD was determined by preincubating 0.2 μg/μL Ct-PtxD protein in 50 mM of aforementioned pH buffers at 25°C for 1 h. Then, 10-µL aliquots were used to determine the residual PtxD activity. For determining the optimal temperature of Ct-PtxD, the reactions were performed under varying temperatures from 30°C to 60°C.

To examine the effect of NAD(P)^+^ binding with Ct-PtxD on thermostability, a solution containing 0.2 μg/μL Ct-PtxD protein in 50 mM MOPS (pH 7.3) was incubated at 45°C after preincubated on ice with or without 0.5 mM NAD(P)^+^ for 5 min. At different times points, a 10-µL aliquot was taken and the residual activity was measured in a solution containing 0.5 mM NAD^+^, 1.0 mM Pt, and 20 mM MOPS (pH 7.3) at 37°C for 10 min.

### 2.6 Effect of salts and organic solvents on PtxD activities

To investigate the effects of different salts on the PtxD activities, different concentrations of NaCl, CaCl_2_, KCl, NH_4_Cl, and MgCl_2_ were added to the reaction solution containing 20 mM MOPS (pH 7.3), 0.5 mM NAD^+^, 1.0 mM Pt, and 2.0 μg PtxD in a 1-mL reaction volume with varied concentrations of each salt ranging from 0 to 200 mM for divalent cations and from 0 to 1,500 mM for monovalent cations. The activities of the PtxDs were measured using the standard assay described above.

To investigate the organic solvent tolerance of Ct-PtxD and Rs-PtxD proteins, the residual PtxD activities were assayed after incubating the enzymes with different concentrations (0%–50%) of *N,N*-dimethylformamide (DMF), methanol, ethanol, and dimethyl sulfoxide (DMSO) in 50 mM MOPS (pH 7.3) containing 0.2 μg/μL Ct-PtxD or Rs-PtxD preincubated with or without 0.5 mM NAD(P)^+^. Ct-PtxD and Rs-PtxD proteins were incubated with organic solvent solution at 25°C for 6 h.

To determine the effects of organic solvents on enzyme activity, PtxD activities were assayed in a reaction solution containing different concentrations of DMF, methanol, ethanol, and DMSO ranging from 10% to 50% (v/v). The reaction solution contained 20 mM MOPS (pH 7.3), 0.5 mM NAD^+^, 1.0 mM Pt, and 2.0 μg PtxD with the varied concentration of organic solvents in a 1-mL reaction volume. The activities of Ct-PtxD and Rs-PtxD were measured using the standard assays described above.

### 2.7 Analysis of *L*-tert-leucine production

TMP is converted into *L*-tert-leucine (*L*-Tle) via reductive amination using LeuDH in the presence of NH_4_^+^ and NADH. The coupling reaction of LeuDH and PtxD was performed in a solution containing 8 mM TMP, 0.1 mM NAD^+^, 20.0 mM Pt, 1.0 U/mL LeuDH, 500 mM NH_4_Cl, and 0.05 U/mL Ct-PtxD or Rs-PtxD in 20 mM Tris-HCl (pH 8.0). The final volume of reaction solutions (200 µL) was incubated at 37°C. L-tert-leucine was determined by HPLC upon derivatization with 2,4-dinitrofluorobenzene (DNFB) as previously described, with minor modifications (Tajima et al., 2015). Briefly, 25 μL of the reaction sample was periodically removed from the reaction solution, mixed with 10 μL 1 M NaHCO_3_ and 40 μL DNFB (37.6 mM in acetone) and incubated at 40°C for 90 min. The reaction was stopped with 10 μL 1 N HCl, then the samples were diluted with 800 μL 20% acetonitrile. The derivatized *L*-tert-leucine were obtained using an HPLC system (Jasco, Tokyo, Japan) fitted with a UV detector and reverse phase column ODS-80TM (Tosoh, Tokyo, Japan) at 360 nm. *L*-Tle was eluted at 40°C using 0.095% trifluoroacetic acid in MilliQ water as mobile phase A and 80% acetonitrile containing 0.1% trifluoroacetic acid as mobile phase B at a flow rate of 1 mL/min for 55 min; 31.3%–48.8% phase B gradient was applied. In this system, the retention time of *L*-Tle was approximately 54.0 min.

### 2.8 Structural analysis

A molecular modeling analysis of Rs-PtxD structure was performed using the PDB: 6IH2 structure (Liu et al., 2019) as a template. The three-dimensional (3D) structure of Ct-PtxD was predicted using Google Colab AlphaFold2 (Jumper et al., 2021). The structures of Rs-PtxD and Ct-PtxD, electrostatic surface potentials, and hydrophobic surfaces were visualized using PyMol with default parameters (Baker et al., 2001) and the Adaptive Poisson-Boltzmann Solver (APBS) electrostatics plugin (Dolinsky et al., 2007). The hydrophobic surfaces of Rs-PtxD and Ct-PtxD were colored according to Eisenberg’s scale of hydrophobicity (Eisenberg, 1984). Representations of protein structures were generated using PyMOL software.

## 3 Results and discussion

### 3.1 Cloning, expression, and purification of Ct-PtxD

**FIGURE 1:**
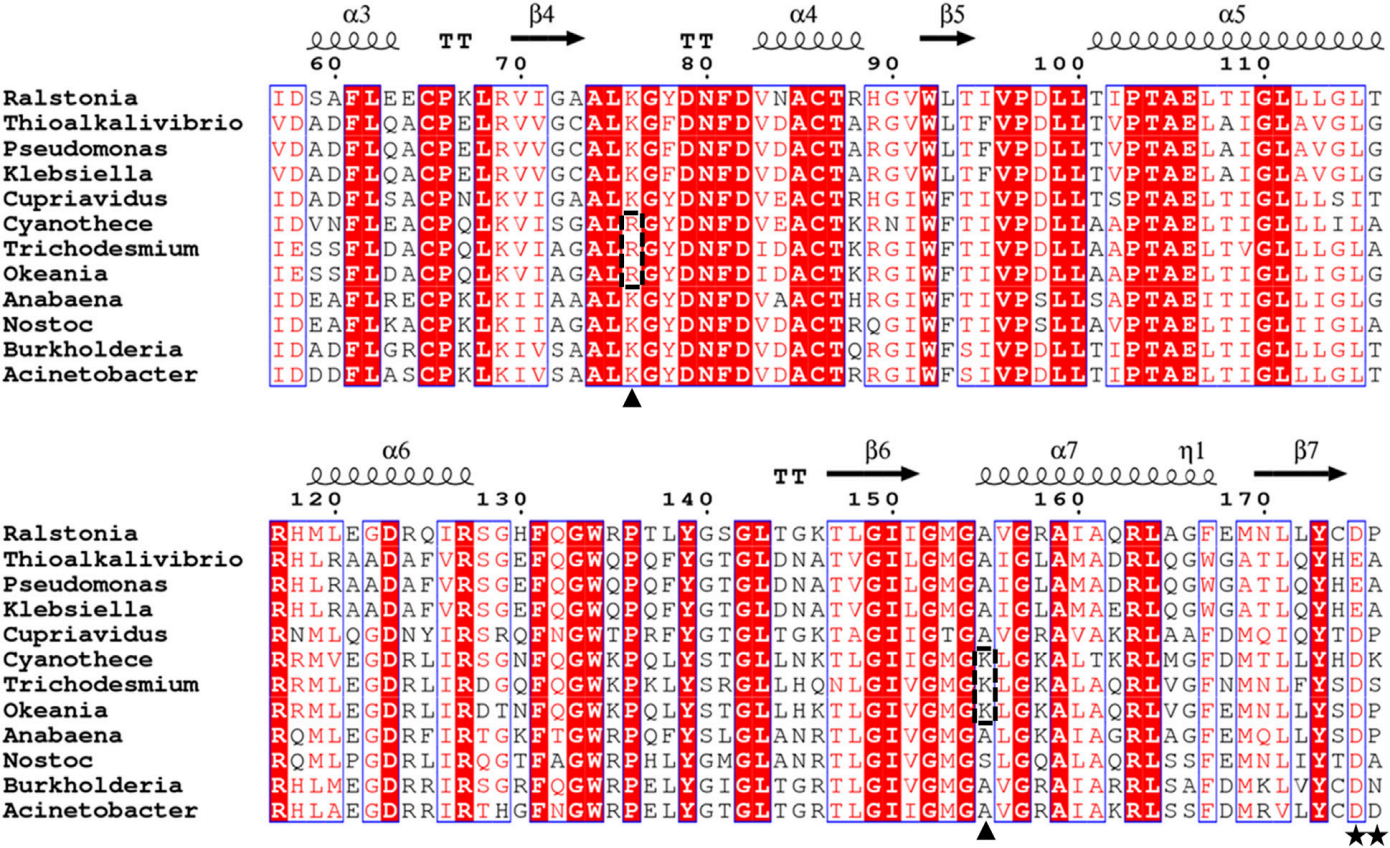
Sequence alignment of PtxDs. The alignment was generated using ESPrint 3.0 (Robert and Gouet, 2014; http://espript.ibcp.fr/ESPript/cgi-bin/ESPript.cgi). The residues responsible for binding the 2′-hydroxyl group of NAD^+^ or 2′-phosphate group of NADP^+^ are indicated by stars, while residues responsible for binding the pyrophosphate group of NAD(P)^+^ that substituted in marine-PtxDs are indicated by arrowheads on the bottom and highlighted with dotted rectangles. The PtxD amino acid sequences used in this analysis are *Ralstonia* sp. 4,506 (BAV60202), *Thioalkalivibrio sulfidiphilus* HL-EbGr7 (ACL72000), *Pseudomonas stutzeri* WM88 (O69054), *Klebsiella pneumoniae subsp. pneumoniae* KPNIH20 (EJK14408), *Cupriavidus basilensis* KF708 (WP_026485313), *Cyanothece* sp. ATCC51142 (WP_009544749), *Trichodesmium erythraeum* GBRTRLIN201 (MBS9771025), *Okeania hirsute* (WP_124144079), Anabaena sp. PCC 7120(WP_010994144), Nostoc sp. KVJ3 (WP_265276617), Burkholderia vietnamiensis G4 (ABO60092), and *Acinetobacter* sp. SH024 (EFF86760).

Alignment of the deduced amino acid sequence of Rs-PtxD with its bacterial counterparts showed significant identity to the sequences of other PtxDs present in GenBank (Figure 1). A 996-bp gene encoding a peptide consisting of 332 amino acids was extracted from the marine cyanobacterium *Cyanothece* sp. ATCC 51142 (Welsh et al., 2008). The deduced amino acid of the gene exhibited 59.8% identity with Rs-PtxD. Thus, we designate the PtxD as Ct-PtxD. Compared to the amino acid sequences of other PtxD proteins, Ct-PtxD possessed fewer hydrophobic amino acids, which are known to be related to salt resistance of the protein (Supplementary Table S1). In addition, some marine-derived enzymes retain high catalytic activity in organic solvents and high salinity (Fuciños et al., 2012), suggesting the potential of Ct-PtxD to be active under organic solvents and salt conditions.

To characterize its enzymatic properties, the Ct-PtxD gene fragment was inserted into the expression vector pET-21b (+), and the recombinant Ct-PtxD was purified via Ni-NTA affinity chromatography. SDS-PAGE analysis showed that the purified protein reached electrophoretic purity with an apparent molecular weight of 40.8 kDa, which was consistent with the theoretical molecular weight of Ct-PtxD (Supplementary Figure S2).

### 3.2 Specific activity and kinetic analysis of Ct-PtxD

The specific activity of the purified Ct-PtxD was similar to that of Rs-PtxD when measured at 37°C using NAD^+^ as a cofactor (Supplementary Figure S3). However, when NADP^+^ was used as a cofactor, Ct-PtxD exhibited specific activity approximately 10-fold higher than that of Rs-PtxD. The kinetic values of Ct-PtxD protein were determined when either NAD(P)^+^ or Pt was saturated. The Ct-PtxD protein exhibited the *K*_M_^NAD^ and *k*_cat_ of 9.07 × 10^−6^ ± 1.05 × 10^−6^ M and 2.24 ± 0.15 s^-1^, respectively, resulting in the catalytic efficiency (*k*_cat_/*K*_M_^NAD^) of approximately 2.5 × 10^5^, which is comparable to that of Rs-PtxD (Table 1; Supplementary Figure S4). In contrast, kinetic analysis with NADP^+^ revealed that Ct-PtxD showed lower *K*_M_^NADP^ and higher *k*_cat_ values than Rs-PtxD, resulting in approximately 12-fold greater catalytic efficiency (*k*_cat_/*K*_M_^NADP^) of Ct-PtxD (Table 1; Supplementary Figure S4). The higher affinity of Ct-PtxD for NADP^+^ than that of Rs-PtxD is likely due to the presence of basic lysine at position 176, which might create a positively charged region that can accept the 2′-phosphate group of NADP^+^ (Figure 1) (Woodyer et al., 2003).

**TABLE 1:** Kinetic parameters of Ct-PtxD and Rs-PtxD^a^.

PtxD	NAD^+^				NADP^+^				References
	*K*_M_ (M)	*k*_cat_ (s^−1^)	*k*_cat_/*K*_M_ (M^−1^ s^−1^)	*K*_M_ (M, Pt)	*K*_M_ (M)	*k*_cat_ (s^−1^)	*k*_cat_/*K*_M_ (M^−1^ s^−1^)	*K*_M_ (M, Pt)	
Ct-PtxD	9.07 × 10^−6^ ± 1.05×10^−6^	2.32 ± 0.18	2.56 × 10^5^	1.68 × 10^−4^ ± 0.25 × 10^−4^	1.22 × 10^−4^ ± 0.04 ×10^−4^	1.02 ± 0.11	8.33 × 10^3^	4.83 × 10^−4^ ± 0.06 × 10^−4^	This study
Rs-PtxD	7.67 × 10^−6^ ± 2.27 × 10^−6^	2.12 ± 0.28	2.76 × 10^5^	3.69 × 10^−5^ ± 6.95 × 10^−6^	4.18 × 10^−4^ ± 0.39 × 10^−4^	0.27 ± 0.11	6.38 × 10^2^	3.38 × 10^−4^ ± 0.41 × 10^−4^	Abdel-Hady et al., 2021

^a^ All assays were performed three times in 100 mM MOPS, at 37°C, pH 7.3.

### 3.3 Biochemical characterization of Ct-PtxD

**FIGURE 2:**
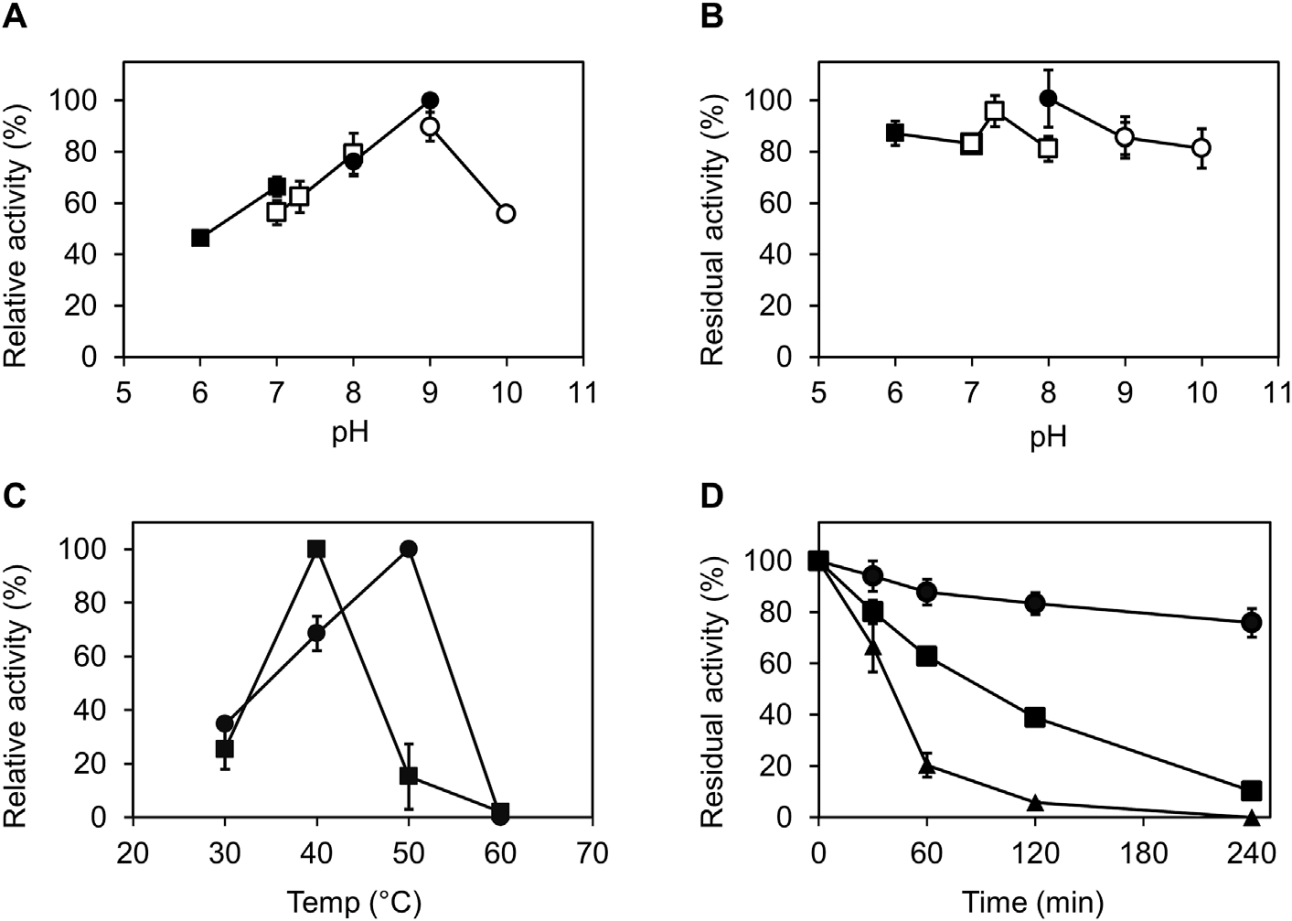
Characterization of Ct-PtxD with respect to pH and temperature. **(A)** Optimal pH for Ct-PtxD activity. The activity was measured using the buffers MES (pH 6.0–7.0; filled squares), MOPS (pH 7.0–8.0; open squares), Tris (pH 8.0–9.0; filled circles), and CHES (pH 9.0–10.0; open circles). **(B)** Effect of pH on the stability of Ct-PtxD activity. The enzyme was preincubated in various pH conditions at 25°C for 1 h, and the residual activity was determined. **(C)** Optimal temperature of Ct-PtxD activity using NAD^+^ (circles) and NADP^+^ (squares) as a cofactor. **(D)** Thermal inactivation of Ct-PtxD at 45°C pre-bound with NAD^+^ (circles), NADP^+^ (squares), and without cofactor (triangles). The enzyme was incubated at 45°C for 6 h, and the remaining activity was measured at different time points at 37°C as described in Material and Methods. The data are shown as means ± standard deviation obtained from three independent experiments.

The activity of the Ct-PtxD protein was evaluated within the pH range of 6.0–10.0. Generally, Ct-PtxD exhibited activity and stability over a wide pH range (6.0–10.0). In Tris-HCl buffer, Ct-PtxD exhibited the highest activity at pH 9.0 (Figure 2A). The optimal pH in the present study was higher than those previously reported for Rs-PtxD (pH 7.3) and Ps-PtxD (pH 7.3) (Costas et al., 2001; Hirota et al., 2012). Moreover, the enzyme retained more than 80% of its optimum activity after incubating in the solutions whose pH values were ranged from 6.0 to 10.0 at 25°C for an hour (Figure 2B), showing its high stability in wide pH ranges compared to other PtxDs.

To determine the optimum temperature of Ct-PtxD, enzyme assays were performed at different temperatures ranging from 30°C to 60°C. When NAD^+^ was used as a cofactor, Ct-PtxD activity increased linearly from 30°C to 50°C and displayed the highest activity at 50°C, whereas Ct-PtxD activity was the highest at 40°C when NADP^+^ was used as a cofactor (Figure 2C). Next, the thermostability of Ct-PtxD protein was tested at 45°C with or without the pre-binding of nicotinamide cofactors during the heat treatment. The thermostability of Ct-PtxD decreased significantly when the protein was heat-treated without the pre-binding of a cofactor. However, preincubating Ct-PtxD with 0.5 mM NADP^+^ increased its thermostability. Moreover, preincubating with 0.5 mM NAD^+^ significantly increased thermostability and kept the residual activity of Ct-PtxD at approximately 80% after thermal inactivation for 4 h (Figure 2D). Previously, we showed that the high NADP^+^ affinity of the Rs-PtxD_HARRA_ mutant provides the enzyme protection from thermal inactivation at 45°C for 6 h (Abdel-Hady et al., 2021). Our observations indicate that when enzymes bind with their preferred cofactors, they form a more thermally stable enzyme–substrate complex, possibly due to the formation of new hydrogen bonds, electrostatic or hydrophobic interactions that stabilize the native protein structure and elevate unfolding temperature. These results imply that Ct-PtxD has excellent operational stability at elevated temperatures when bound to nicotinamide cofactors.

### 3.4 Effect of salt ions on the activity of PtxD proteins

**FIGURE 3:**
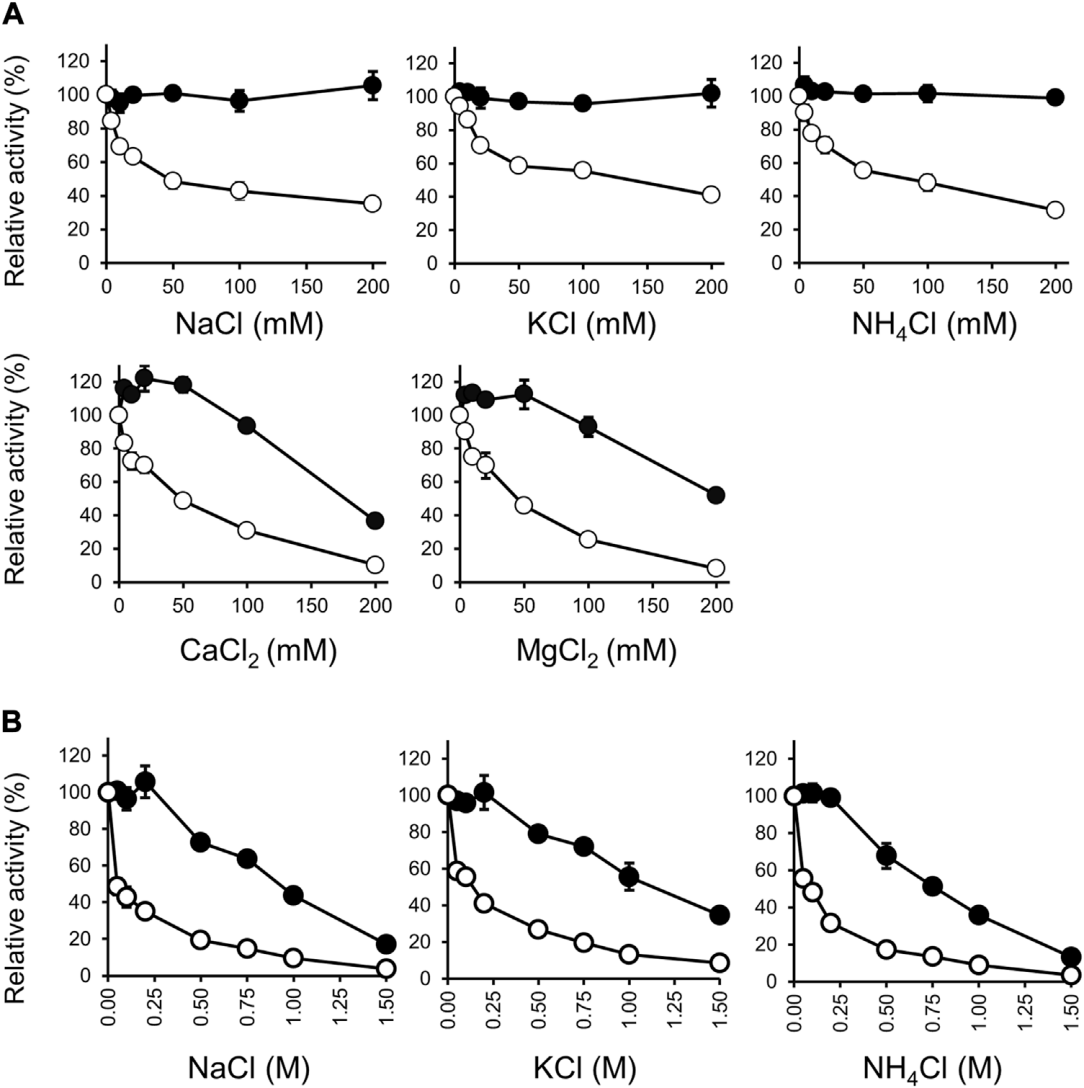
Effect of salts on the activities of Ct-PtxD (closed circle) and Rs-PtxD (open circle). **(A)** The activities were measured at 37°C with different salts up to 200 mM added to the reaction solution separately. **(B)** Effect of NaCl, KCl, and NH_4_Cl on the activities measured at 37°C with different concentrations up to 1.5 M added to the reaction solution separately. Incubation of PtxD enzymes in the absence of added reagents was the control treatment. The values are expressed as averages of triplicate and the error bars mean standard deviation.

Salt ions had different effects on the activity of Rs-PtxD and Ct-PtxD proteins (Figure 3). For Rs-PtxD enzyme, the monovalent cations, such as Na^+^, K^+^ and NH_4_^+^, gradually inhibited the activity of the enzyme from 4 to 200 mM, whereas more inhibition was observed with the divalent cations Ca^2+^ and Mg^2+^ even under lower concentrations ranged from 4 to 20 mM (Figure 3A). Notably, the monovalent cations Na^+^, K^+^, and NH_4_^+^ exhibited no significant effect on the activity of Ct-PtxD enzyme from 0 to 200 mM, whereas the divalent cations Ca^2+^ and Mg^2+^ caused approximately 60% and 40% inhibition of the activity at only higher concentration (200 mM), respectively (Figure 3A). Despite salt tolerance being relevant at high concentrations, the divalent cations Ca^2+^ and Mg^2+^ might be useful at only lower concentrations (up to 10 mM) for NAD(P)H cofactor regeneration (Tamura et al., 1990), meaning Ct-PtxD might be more effective than Rs-PtxD in reactions required lower concentration of Ca^2+^ and Mg^2+^.

We investigated the effects of monovalent cations Na^+^, K^+^, and NH_4_^+^ on the activities of Rs-PtxD and Ct-PtxD at concentrations up to 1.5 M (Figure 3B). All monovalent cations inhibited approximately 90% of Rs-PtxD enzyme activity at a concentration of 1.0 M. However, Ct-PtxD retained approximately 50% of its activity with both Na^+^ and K^+^, and 36% of activity was observed with NH_4_^+^ at 1.0 M concentration. This type of salt resistance has not been reported in other PtxDs, including Ps-PtxD (Costas et al., 2001), possibly because the Ct-PtxD originates from a bacterium thriving in marine environments (Ghattavi and Homaei, 2023). Some proteases were isolated from marine bacteria with thermostability, salt tolerance, and solvent stability (Marcello et al., 1996; Oda et al., 1996; Ogino et al., 1999). Thus, these results indicate that Ct-PtxD may be suitable for NADH regeneration under high-salt conditions.

### 3.5 Effect of organic solvents on stabilities and activities of PtxDs

**FIGURE 4:**
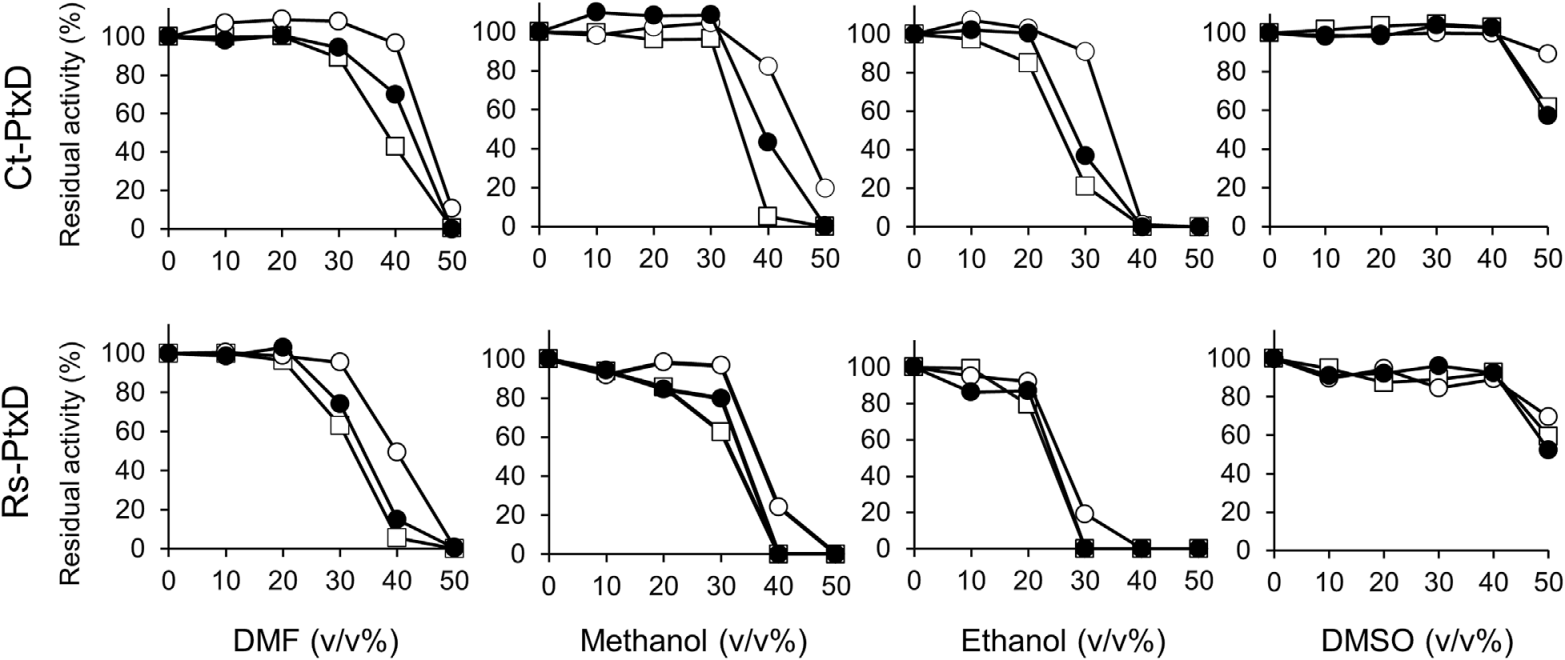
Characterization of the tolerance to organic solvents for Ct-PtxD and Rs-PtxD enzymes. Prior to the assay reaction, PtxD enzymes were incubated in the 50 mM MOPS (pH 7.3) containing 0.5 mM NAD^+^ (open circles), 0.5 mM NADP^+^ (closed circles), or without cofactor (squares). The reactions were performed by using 0.5 mM NAD^+^ as a cofactor. The data are representative of two independent experiments.

The high salt resistance of enzymes is often positively correlated with organic solvent resistance because underlying resistance mechanisms of both are similar in terms of the management of reduced water activity (Sellek and Chaudhuri, 1999). Sana et al. (2006) reported that a salt protease from a gamma proteobacterium isolated from a marine environment is stable in the presence of various solvents. Generally, Ct-PtxD is relatively stable in the presence of organic solvents compared to Rs-PtxD (Figure 4). Without cofactor preincubation, Ct-PtxD maintained about 84%–97% of its activity after 6 h of incubation in 30% DMF or methanol and approximately 21% activity at the same concentration of ethanol (Figure 4). In contrast, Rs-PtxD retained only 60% of its activity at the same concentration of DMF or methanol, and completely lost its activity under ethanol conditions. These results indicate that Ct-PtxD is more stable in these organic solvents than Rs-PtxD. After preincubating Ct-PtxD with 0.5 mM NAD^+^ before incubating in 40% of DMF or methanol, and 30% ethanol (v/v) for 6 h, the enzyme almost completely retained its total residual activity (Figure 4). However, Rs-PtxD incubated with the organic solvents at the same concentration showed only 20%–50% of its activity (Figure 4). Likewise, preincubating with 0.5 mM NADP^+^ before incubation with 40% DMF or methanol, and 30% ethanol, Ct-PtxD showed 40%–70% of the residual activity. In contrast, Rs-PtxD lost its activity or retained only 15% of its total activity in the same concentration of organic solvents. DMSO had no significant effect on enzymes preincubated with or without cofactors. These data strongly indicate that Ct-PtxD was tolerant to these organic solvents.

**FIGURE 5:**
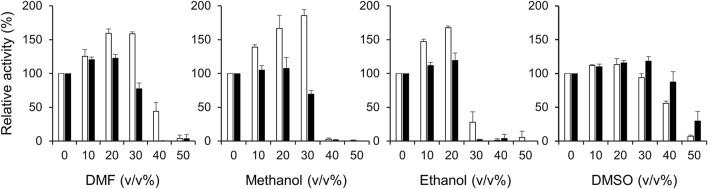
Effect of organic solvents on the activities of Ct-PtxD (open columns) and Rs-PtxD (closed columns) enzymes. The activities were measured at 37°C with different concentrations of organic solvents added to the reaction solution. The reactions were performed using 0.5 mM NAD^+^ as a nicotinamide cofactor. The values are expressed as averages of triplicate and the error bars mean standard deviation.

Next, we investigated the activities of Ct-PtxD and Rs-PtxD in the presence of organic solvents in a reaction mixture (Figure 5). Remarkably, the Ct-PtxD activity enhanced in the reaction solution containing most tested organic solvents up to 30% (v/v). In the case of 20%–30% volume of DMF in the reaction solution, Ct-PtxD activity enhanced by approximately 160%. The addition of methanol or ethanol showed the same trends on the Ct-PtxD activity, and the activity was enhanced up to approximately 185% by adding 30% methanol (v/v). In contrast, although Rs-PtxD activity was slightly enhanced by the addition of 20% of DMF and ethanol, the effects were not significant and increased concentration of all tested solvents (>30%) strongly inhibited its activity. DMSO did not show this enhancement effect, probably because its higher solubility in water correlated with its lower log *P*_ow_ value (−1.35) (Li and Yu, 2012). In contrast, Cui et al. (2020) reported that the catalytic efficiency (*k*_cat_/*K*_M_) of *Bacillus subtilis* lipase A decreased under 1,4-dioxane, DMSO, and 2,2,2-trifluoroethanol organic solvents. They showed that the *K*_M_ of lipase A increased in the presence of organic solvents than under aqueous conditions. Although the reason for this mechanism is unknown, they concluded that the reduction in catalytic efficiency in selected organic solvents was attributed to decreased turnover (*k*_cat_) and increased *K*_M_ values. Consequently, our results strongly suggest that the improved activity of Ct-PtxD in organic solvents is likely due to the increased *k*_cat_. Overall, these results demonstrate that Ct-PtxD is not only stable but also activates the initial activity in organic solvents.

### 3.6 Ct-PtxD has a positively charged NAD^+^-binding site and low hydrophobic content that contributes to the resistance against salt and organic solvent

**FIGURE 6:**
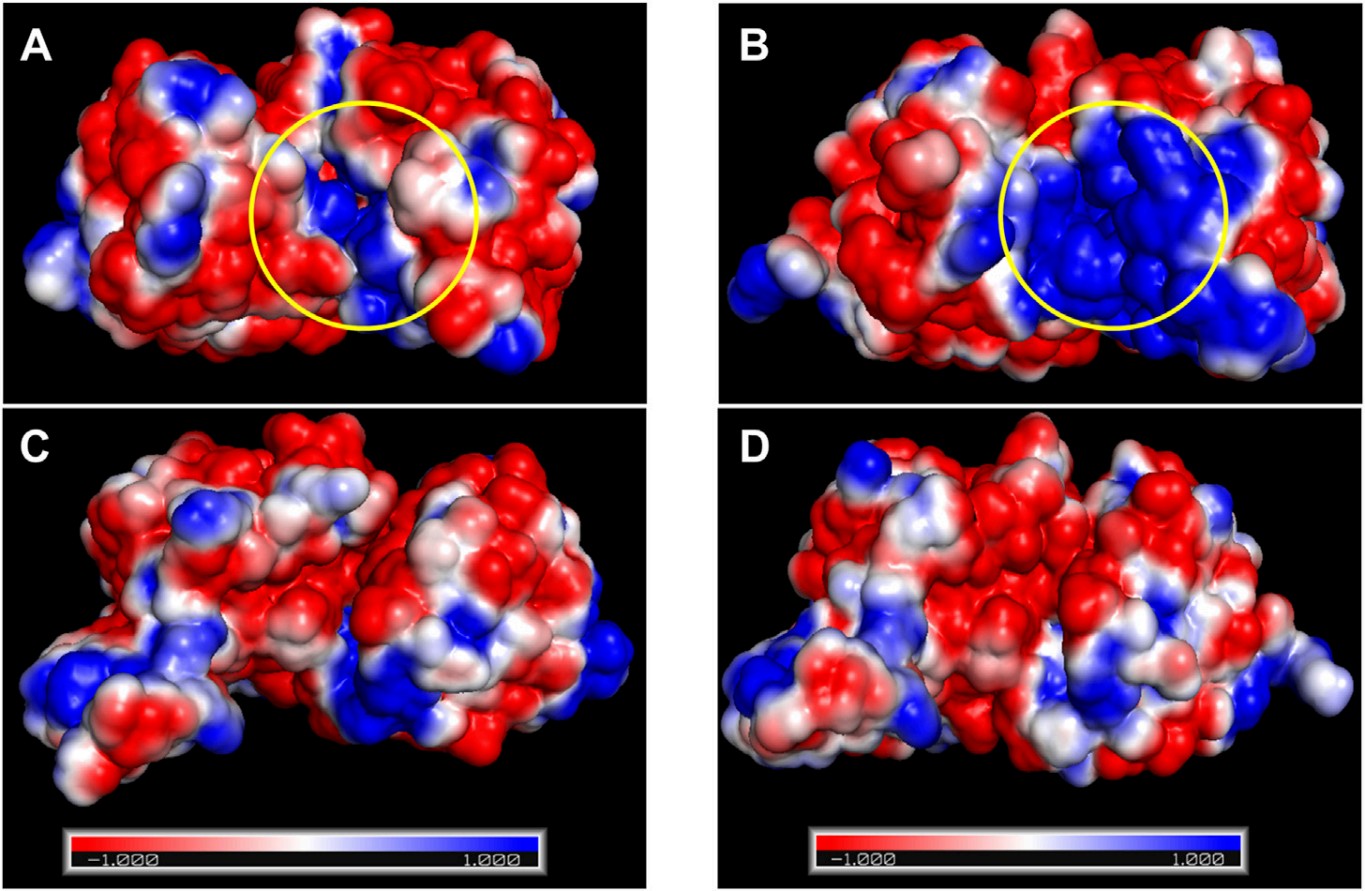
Surface electrostatic potential analysis of Rs-PtxD and Ct-PtxD. **(A)** The surface electrostatic potential of Rs-PtxD. **(B)** The surface electrostatic potential of Ct-PtxD obtained by PyMol and APBS plugin. **(C)** The 90° rotated view of **(A)**. **(D)** The 90° rotated view of **(B)**. The negative and positive residues are indicated by red and blue, respectively. The NAD^+^-binding regions are indicated using a yellow circle.

Electrostatics are known to play a pivotal role in the affinity of proteins for highly charged biological molecules. For instance, most proteins that bind to nucleic acids possess more positively charged residues, particularly in their binding sites, to electrostatically attract the negatively charged backbone of DNA/RNA (Vascon et al., 2020). In the presence of salts, cations electrostatically attract the highly negatively charged surface of the DNA, forming a zone called an ion atmosphere. When proteins bind to nucleic acids, ions are released from the ion atmosphere owing to electrostatic interactions between positively charged protein side chains and negatively charged phosphates (Yu et al., 2020). The surface electrostatic potential analysis suggested that Ct-PtxD is rich in positively charged residues in the binding region of NAD^+^ compared to other PtxD enzymes, which may promote engagement with the negatively charged pyrophosphate group of NAD^+^ under high-salt conditions (Figure 6; Supplementary Figure S5). In addition, in organic solvents, positively charged residues may attract more water molecules and prevent interactions with the organic solvents, resulting in a higher hydration shell and improved NAD^+^ binding (Cui et al., 2020). In most PtxD enzymes, Lys-76 involves favorable electrostatic interactions with the pyrophosphate oxygens of the NAD(P)^+^ cofactor (Zou et al., 2012) (Supplementary Figure S6). Compared to other PtxDs, this residue was substituted with arginine in marine-derived PtxDs, including Ct-PtxD (Figure 1). Compared to Lys, Arg side chains are strongly attracted to negatively charged molecules, partially because the Arg guanidinium groups possess a higher number of nitrogen atoms than the NH_3_^+^ groups in Lys, which are used as hydrogen-bonding donors (Yu et al., 2020). Furthermore, the alanine residue at position 155 is highly conserved in PtxDs and is involved in NAD^+^ binding by forming a hydrogen bond with the pyrophosphate of NAD^+^ (Zou et al., 2012) (Figure 1; Supplementary Figure S6). Notably, Ala 155 of Ct-PtxD was substituted with lysine, of which the side chain might electrostatically attract pyrophosphate group of NAD^+^ more than that of non-polar alanine residue found in Rs-PtxD. Recently, the replacement of alanine 155 with isoleucine was shown to improve the stability and NAD^+^ binding of PtxD owing to an increase in the number of hydrogen bonds formed (Baammi et al., 2023). Taken together, these substitutions may contribute to stronger electrostatic interactions with the NAD^+^ cofactor and higher Ct-PtxD activity in the presence of salts or organic solvents.

As stated in the introduction section, salt- and organic solvent-active enzymes maintain an adequate hydration shell under low water activity. According to previous reports, higher hydration shells at the protein surface can be attributed to a lower hydrophobic amino acid composition, which reduces hydrophobicity and increases water contact with the protein surface (Karan et al., 2012; Wang et al., 2016). Compared to other PtxD, Ct-PtxD displayed a lower percentage of hydrophobic amino acids, which constituted approximately 45.6% (Supplementary Table S1). Based on the 3D structure of Ct-PtxD predicted using AlphaFold2 and the available structure of Rs-PtxD (Protein Data Bank ID: 6IH2; Liu et al., 2019) using PyMol (color-h), the hydrophobic amino acids may be less distributed on the surface of Ct-PtxD than on Rs-PtxD (Supplementary Figure S7). Furthermore, Ct-PtxD had a lower number of alanine (6.6%) and proline (4.5%) residues compared to all other PtxD enzymes (Supplementary Table S1), which may contribute to the reduced hydrophobic surface and increased water interaction with Ct-PtxD (Wang et al., 2016).All of these factors, in combination, might lead to the formation of a solvation shell that keeps the protein surface hydrated, increases the structural stability of Ct-PtxD, and consequently plays a key role in maintaining its high catalytic activity under high salinity and in higher organic solvents.

### 3.7 *L*-Tle production using Ct-PtxD as an NADH-generation system

**FIGURE 7:**
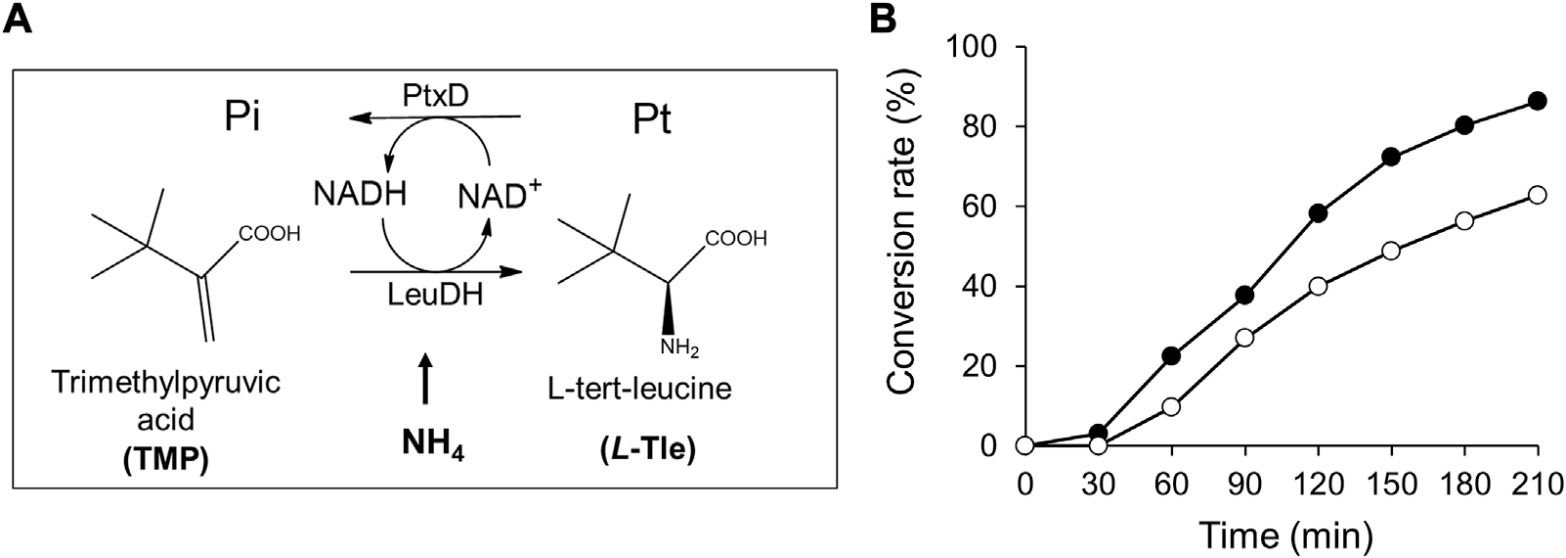
*L*-tert-Leucine (*L*-Tle) production by leucine dehydrogenase with NADH regeneration by Ct-PtxD and Rs-PtxD. **(A)** A schematic of the coupled reactions of *L*-Tle production by leucine dehydrogenase (LeuDH) and NADH regeneration by PtxD. **(B)** The batch production of *L*-Tle from trimethylpyruvic acid (TMP) by LeuDH with NADH regeneration by Ct-PtxD (closed circles) and Rs-PtxD (open circles). The reaction solutions contained 20 mM Pt, 8 mM TMP, 0.1 mM NAD^+^, 1.0 U/mL of LeuDH, and 0.05 U/mL of PtxD proteins.

To demonstrate the advantage of Ct-PtxD as a cofactor regeneration system, the NADH-regeneration reaction of Ct-PtxD and Rs-PtxD was coupled with *L*-Tle production using LeuDH (Figure 7A). LeuDH uses NADH as a cofactor and catalyzes the reductive amination of TMP into *L*-Tle, which is an important building block in the pharmaceutical, cosmetic, and food additive industries (Jia et al., 2021). This reaction involves a high concentration of ammonium ions as an amino group donor, which inhibits PtxD activity (Figure 3). In the *L*-Tle production reaction of LeuDH using PtxD as an NADH-regeneration enzyme, 8 mM TMP was used as the substrate in the presence of 0.1 mM NAD^+^. When Rs-PtxD was used as the regeneration enzyme, only 60% of the substrate conversion was supported, probably because of the sensitivity to high ammonium concentrations (Figure 7B). In contrast, Ct-PtxD supported approximately 84% conversion of TMP into *L*-Tle in 210 min, suggesting that it was effective in the cofactor-regeneration reaction under high concentrations of ammonium.

## Data Availability

The datasets presented in this study can be found in online repositories. The names of the repository/repositories and accession numbers can be found in the article/Supplementary Material.
