# Supplementary material for: A novel salt- and organic solvent-tolerant phosphite dehydrogenase from Cyanothece sp. ATCC 51142
Source: Front Bioeng Biotechnol. 2023 Aug 18;11:1255582. doi: 10.3389/fbioe.2023.1255582 (PMC10473253; doi:10.3389/fbioe.2023.1255582)
Supplement: Supplementary file 1 [file DataSheet1.pdf]

Supplementary Information for

A Novel Salt- and Organic Solvent-Tolerant Phosphite Dehydrogenase from *Cyanothece* sp. ATCC 51142

Gamal Nasser Abdel-Hady[1,2], Takahisa Tajima[1,3], Takeshi Ikeda[1], Takenori Ishida[1], Hisakage Funabashi[1,3], Akio Kuroda[1,3], Ryuichi Hirota[1,3*]

[1] Unit of Biotechnology, Division of Biological and Life Sciences, Graduate School of Integrated Sciences for Life, Hiroshima University, Hiroshima, Japan

[2] Department of Genetics, Faculty of Agriculture, Minia University, Minia, Egypt

[3] Seto Inland Sea Carbon-neutral Research Center, Hiroshima University, Japan

*** Correspondence:**

Ryuichi Hirota

hirota@hiroshima-u.ac.jp

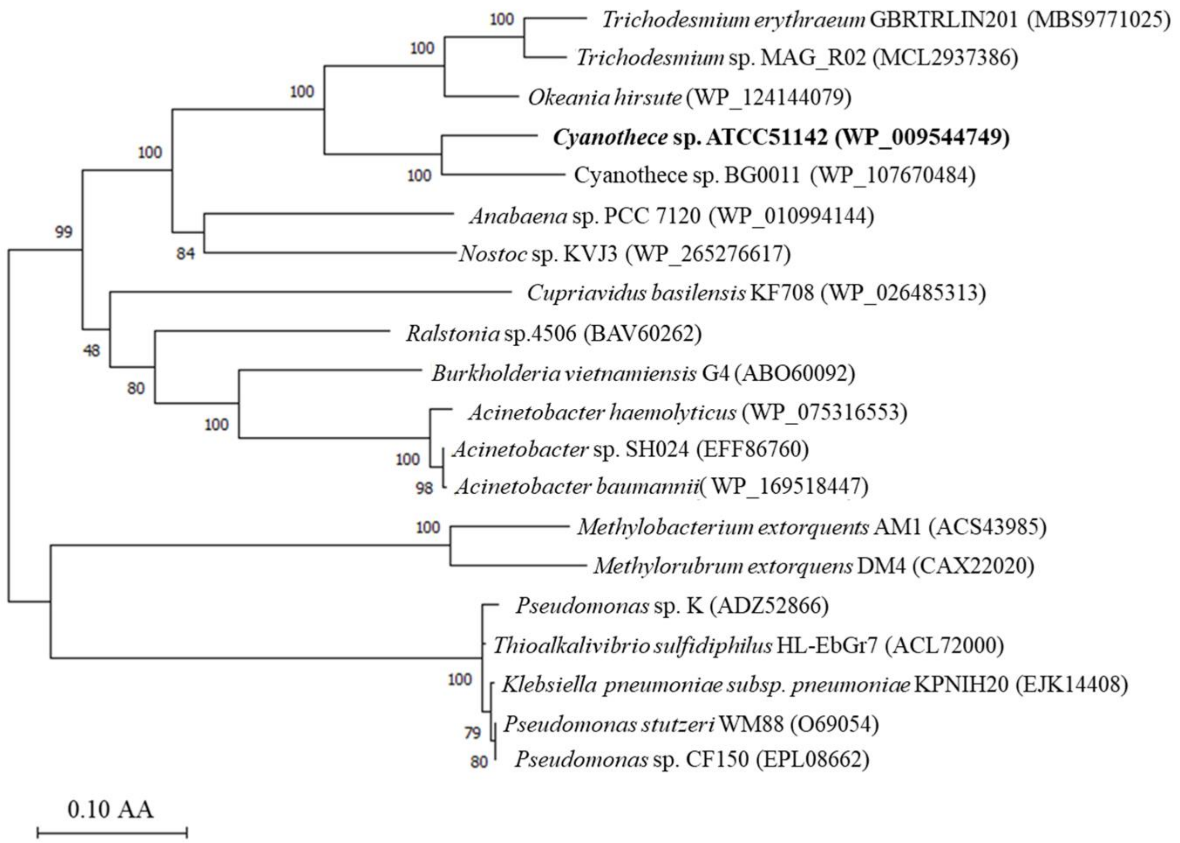

Supplementary Figure 1. Phylogenetic tree of the amino acid sequences of Ct-PtxD and its close homologs. The tree was constructed using the neighbor-joining method (MEGA-X). Bootstrap values (n = 100 replicates) are reported as percentages. The scale bar represents the number of changes per amino acid position. The accession number of each amino acid sequencing is shown in parentheses.

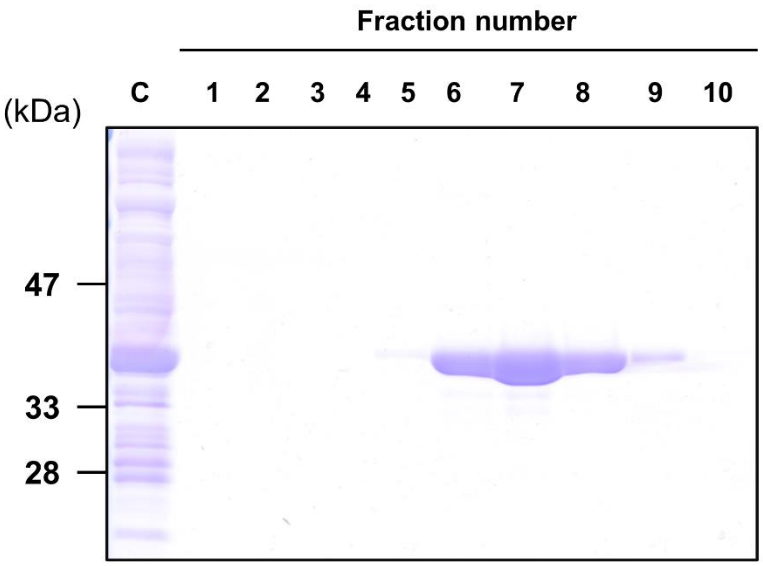

Supplementary Figure 2. SDS-PAGE analysis of the recombinant Ct-PtxD protein purified using a nickel column. Indicated fractions were collected, buffer exchanged, and concentrated for the kinetic analysis. C: crude extract.

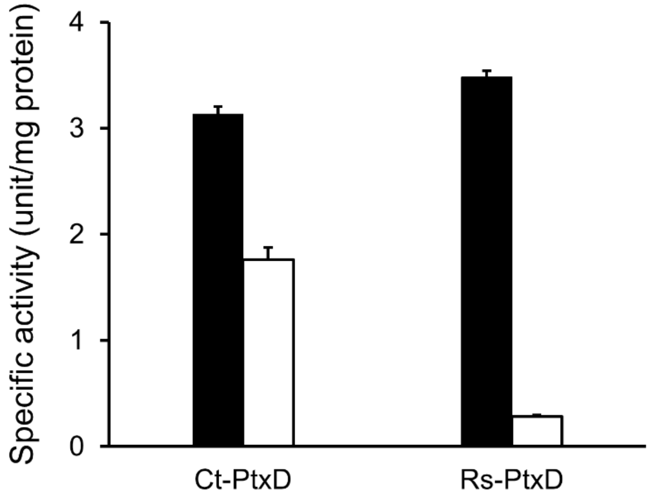

Supplementary Figure 3. The activity of Ct-PtxD and Rs-PtxD measured in one milliliter reaction mixture contained 20 mM morpholinepropanesulfonic acid (MOPS, pH 7.3), 0.5 mM NAD(P)$^+$ (closed columns) or 0.5 mM NADP$^+$ (open columns), and 2.0 μg of PtxD preincubated at 37°C for 2 min. Then, the reaction was started by adding Pt to the mixture at a final concentration of 1.0 mM and running for 10 min at 37°C. Initial velocities were determined by measuring the production of NAD(P)H at 340 nm and calculated using the extinction coefficient of 6,220 M-1 cm-1 (Bucher et al., 1974). One enzyme unit was defined as the amount of enzyme that catalyzed the formation of 1 μmol of NAD(P)H per minute.

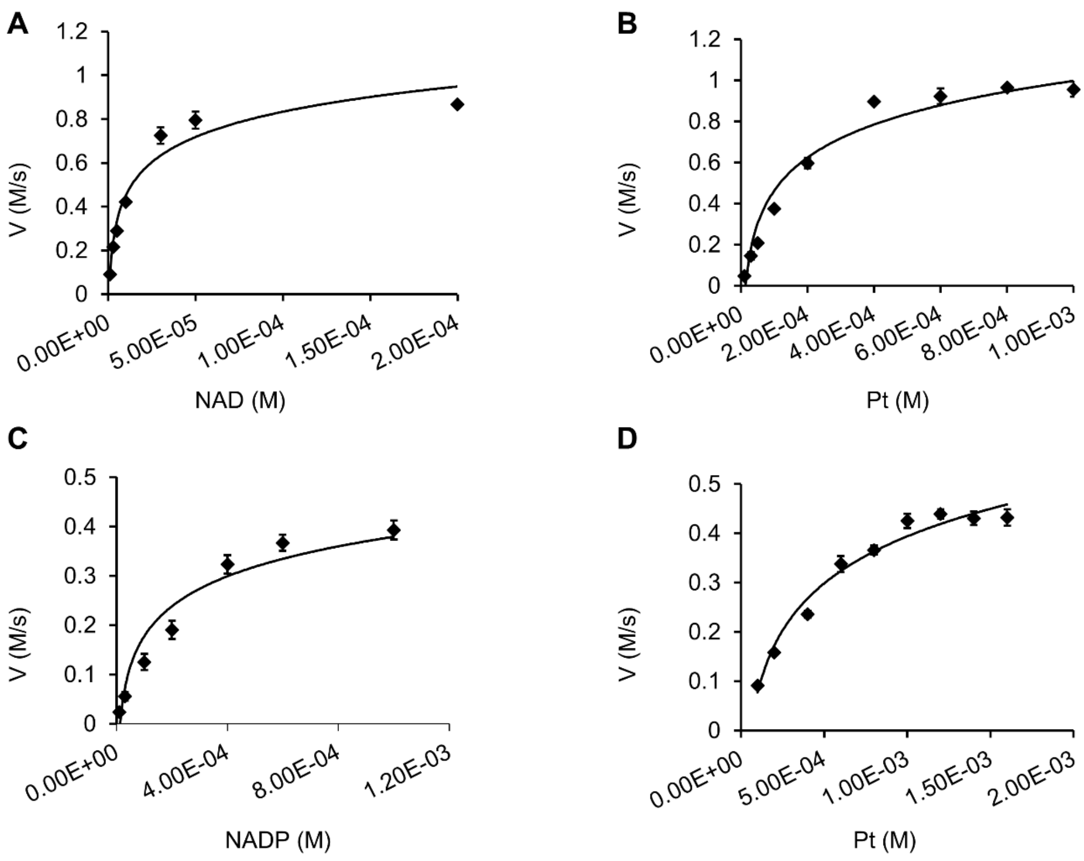

Supplementary Figure 4. Steady state kinetics plots of Ct-PtxD conducted in solutions containing Pt, $NAD(P)^+$, 5.0 µg/mL Ct-PtxD protein, and 100 mM MOPS (pH 7.3) at 37°C. **A)** Initial velocity with NAD and saturated Pt concentration (1.0 mM). **B)** Initial velocity with Pt and saturated NAD concentration (1.0 mM). **C)** Initial velocity with NADP and saturated Pt concentration (1.0 mM). **D)** Initial velocity with Pt and saturated NADP concentration (1.0 mM).

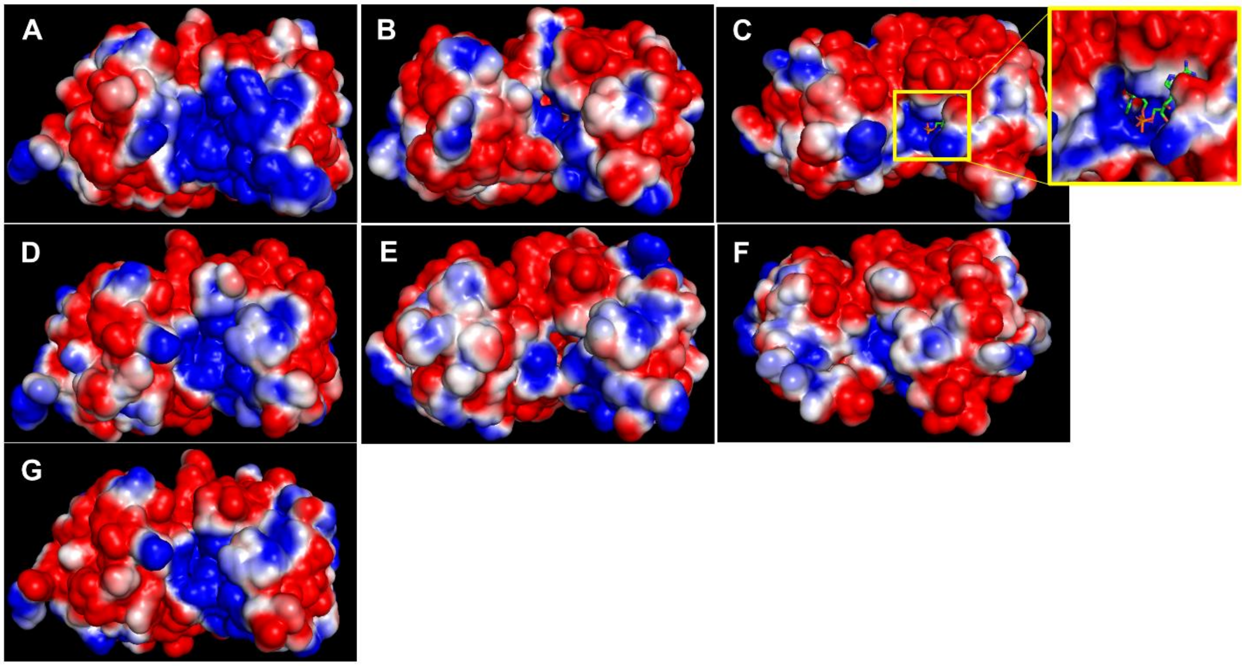

Supplementary Figure 5. Electrostatic surface analysis of different PtxD from (A) *Cyanothece* sp. ATCC51142, (B) *Ralstonia* sp. 4506, (C) *Pseudomonas stutzeri* WM88, (D) *Okeania hirsute, (E) Cupriavidus basilensis* KF708, (F) *Burkholderia vietnamiensis* G4, (G) *Trichodesmium erythraeum* GBRTRLIN201. Close-up view of the NAD binding pocket with NAD cofactor of Ps-PtxD (PDB: 4E5N, Zou et al., 2012) are noted in yellow box.

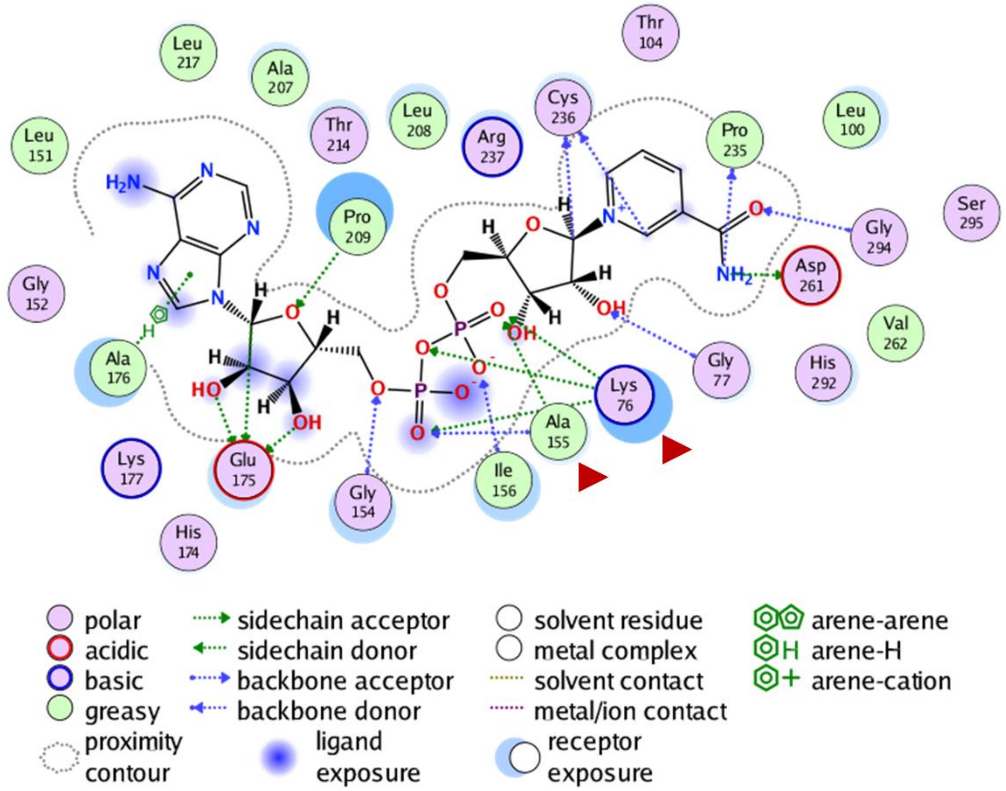

Supplementary Figure 6. Molecular interaction of NAD ligand with PtxD from *Pseudomonas stutzeri* WM88 (Ps-PtxD) (PDB: 4E5N, Zou et al., 2012). The residues Lys76 and Ala155 forming H-bonding with pyrophosphate group are indicated with red arrow heads.

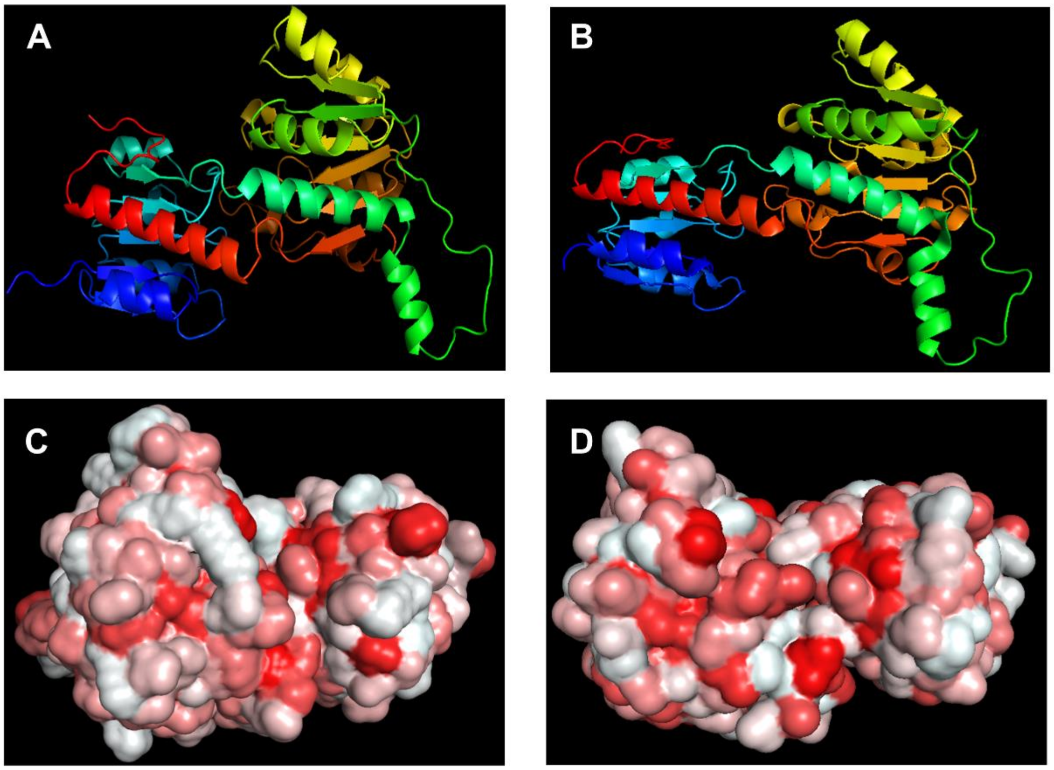

Supplementary Figure 7. Structure and hydrophobic surface analysis of Ct-PtxD and Rs-PtxD. (A) Homology model structure of Ct-PtxD predicted with Google Colab AlphaFold2 (Jumper et al. 2021, Nature). (B) Structure of Rs-PtxD(PDB: 6HI2; Liu et al., 2019) (C) The hydrophobic surface of Ct-PtxD. (D) The hydrophobic surface of Rs-PtxD. The hydrophobic surface obtained by PyMol and APBS plugin, hydrophobic residues are indicated with (red gradient).

Supplementary Table 1. Comparison of the amino acid compositions of Ct-PtxD and other PtxD enzymes [a]

**PtxD (number of amino acids)**	**Ct-PtxD (332)**	**Rs-PtxD (336)**	**Ps-PtxD (336)**	**Ana-PtxD (332)**	**Burk-PtxD (332)**	**Cb-PtxD (333)**
**Hydrophobic aa (%)**	**45.6**	50.6	52.5	49.2	48	46.2
**Ala percent (%)**	**6.6**	12.8	14.9	10.2	9.4	10.5
**Pro percent (%)**	**4.5**	5.7	5.1	6.6	5.6	6.0
**Acidic aa (%)**	**11.4**	11.6	11.6	11.1	12.4	11.7

a. Ana-PtxD from *Anabaena* sp. PCC 7120, Burk-PtxD from *Burkholderia vietnamiensis* G4, and Cb-PtxD from *Cupriavidus basilensis* KF708. The accession numbers for each protein have been shown in Figure 1.
